# Clinical evaluation of pulmonary hypertension using patient-reported outcomes: a cross-sectional study

**DOI:** 10.1186/s12890-021-01416-7

**Published:** 2021-02-02

**Authors:** Miguel Ángel Amor-García, Sara Ibáñez-García, Xandra García-González, Teresa Mombiela, Cristina Villanueva-Bueno, Ana Herranz-Alonso, María Sanjurjo-Sáez

**Affiliations:** 1 Pharmacy Service, Hospital General Universitario Gregorio Marañón, Instituto de Investigación Sanitaria Gregorio Marañón, Doctor Esquerdo, 46, 28007 Madrid, Spain; 2 Department of Cardiology, Hospital General Universitario Gregorio Marañón, Instituto de Investigación Sanitaria Gregorio Marañón, Madrid, Spain

**Keywords:** Pulmonary hypertension, Quality of life, Health-related quality of life, CAMPHOR, Pharmacy services

## Abstract

**Background:**

Patients with pulmonary hypertension (PH) have progressive and disabling symptoms, as well as a burden of treatments and a difficult clinical evaluation that make health-related quality of life a particularly relevant endpoint in this disease. The objective of the study was to evaluate patient-reported outcomes of patients receiving specific treatment for PH in a tertiary hospital using a specific questionnaire (Cambridge Pulmonary Hypertension Outcome Review-CAMPHOR) in the pharmacy consultation.

**Methods:**

A cross-sectional, observational, descriptive study was conducted. It included all patients receiving specific treatment for PH in a tertiary hospital in Madrid, Spain. The inclusion period comprised between August to December 2019. CAMPHOR questionnaires containing three domains: symptoms, activities and quality of life were completed by the patients at the pharmacy consultation. Demographic and clinical variables, including WHO Functional Class (WHO FC), PH-specific tests and hemodynamic parameters, were recorded. Non-parametric analyses to assess relations between variables and CAMPHOR domains were performed.

**Results:**

Thirty-six patients consented to participate in the study and completed the questionnaire. Median scores for symptoms, activities, and quality of life domains were 5.5 (2.5–10), 8.0 (4.5–10.5) and 3.5 (1–7.5), respectively. Statistically significant differences were found in the three domains when comparing by WHO FC, in the activities domain for 6-m walking test and in the quality of life domain for patients who had emergency visits or hospitalizations in the last year.

**Conclusions:**

The CAMPHOR questionnaire could be useful as a complementary test to achieve an integrated evaluation of PH patients, who could complete it easily during their routine pharmacy visits.

## Background

Pulmonary hypertension (PH) is a term that comprises a variety of diseases consisting of elevated blood pressure in the pulmonary circulation. The diagnosis is determined by a resting pulmonary arterial pressure (PAPm) of ≥ 20 mmHg [1]. The most recent classification of PH comprises group 1—pulmonary arterial hypertension (PAH), group 2—PH due to left heart disease (LHD), group 3—PH due to lung diseases and/or hypoxia and group 4—chronic thromboembolic pulmonary hypertension (CTEPH) and group 5- unknown mechanism/multifactorial [2].

The clinical evaluation of these patients is made using variables such as World Health Organization functional class (WHO FC), 6-min walking distance (6MWD), N-terminal prohormone of brain natriuretic peptide (NT-proBNP), cardiac index (CI) and right atrial pressure (RAP) [3, 4]. However, more than one variable is usually required because no single one provides enough diagnostic and prognostic information, also they do not provide information about the health status and quality of life (QoL) of patients [2, 5].

PH produces progressive, disabling symptoms that increase morbidity and mortality [6]. These symptoms (including shortness of breath, fatigue, chest pain, and lightheadedness) have a big impact on WHO FC and emotional state which adversely affects health-related quality of life (HRQoL) [7, 8]. Also, these patients need to use specific treatments: phosphodiesterase type 5 inhibitors (PDE-5 is), endothelin-receptor antagonists (ERA), soluble guanylate cyclase stimulators (sGCs), prostacyclin analogs or prostacyclin receptor agonists (PCa and PCra) [9]. These treatments are associated with very frequent adverse events (AE) including headache, flushing, and epistaxis for PDE-5 is (sildenafil and tadalafil); hepatotoxicity, peripheral edema, and anemia for ERA (bosentan, ambrisentan, and macitentan); and dizziness or hypotension for sGCs (riociguat). Finally, PCa and PCra (epoprostenol, treprostinil, iloprost, and selexipag) are associated with gastrointestinal symptoms, headache, and jaw pain [10]. These treatment-related AE together with the inconvenience of some routes of drug administration (IV, subcutaneous or inhaled especially) can negatively influence a patient´s daily life [11].

Difficulties in clinical evaluation, the impact of symptoms, and the burden of treatments make HRQoL a particularly relevant endpoint in PAH [12]. The importance of HRQoL has been well established to define patient-reported outcomes (PRO´s) as a report of the status of a patient´s health condition that comes directly from the patient, without interpretation of the patient´s response by any health professional [13]. The first instrument designed to assess PRO´s in PH patients was the Cambridge Pulmonary Hypertension Outcome Review (CAMPHOR) [14]. This questionnaire has shown to be valid, reliable and responsive, and is recommended for use alongside traditional clinical measures, also, it has obtained interesting results compared to generic questionnaires such us Nottingham Health Profile (NHP), EuroQoL or SF-36 [15, 16].

The objective of our study was to evaluate PRO´s of patients receiving specific treatment for PH in a tertiary hospital using CAMPHOR in the pharmacy consultation.

## Methods

### Study design

This is a cross-sectional, observational, descriptive study of all patients receiving specific treatment for PH in a tertiary hospital in Madrid, Spain. The inclusion period comprised between August to December 2019.

During the routine follow-up, patients were asked to participate in the study at any pharmacy visit. CAMPHOR was completed by the patients at the pharmacy consultation using a pen and paper version which was previously printed in a booklet format.

Study inclusion criteria were patients with age > 18 years, undergoing a specific treatment for PH in our hospital, Spanish-speakers, able to read and understand the questionnaire, and able to provide informed consent. Exclusion criteria included patients who were not able to answer the questions of CAMPHOR by themselves (due to cognitive impairment or other reasons) or those who completed CAMPHOR by telephone.

Demographic data (age and gender) and clinical data (WHO FC, time since PH diagnosis, PH etiology, time since starting of the current treatment, type of specific therapy for PH, line of treatment, concomitant drugs -including adjuvant treatments for PH-, emergency visits (EV) and hospital admissions (HA) in the 12 months before the inclusion in the study) were compiled. Other variables related to PH such as 6MWD, NT-proBNP, and hemodynamic parameters measured by right heart catheterization (RHC) were also recorded. Patient and treatment data were obtained from the HCIS® electronic medical records and Farhos® CPOE (Computerized Physician Order Entry) software.

### Cambridge pulmonary hypertension outcome review (CAMPHOR)

CAMPHOR was the first questionnaire specifically designed for PH patients. It contains 3 domains: symptoms, activities, and quality of life. The symptoms (impairments) domain contains 25 negatively weighted items related to energy, breathlessness, and mood. Each item has a "yes" or "no" answer scored as 1 or 0, respectively, added to give a total score which can range from 0 to 25. The activity (disability) domain consists of a 15 item scale, rated by patients as being able to perform each activity: on their own without difficulty (scored 0), able to do on own with difficulty (scored 1) or unable to do on own (scored 2). Finally, the quality of life domain contains also 25 negatively weighted items, scored using the same method as for the symptoms score. The theoretical basis for the CAMPHOR is the needs-based model of quality of life which postulates that life gains its quality from the ability and capacity of the individual to satisfy his or her human needs. Higher scores in the different domains of CAMPHOR indicate poor outcomes [15]. A validated Spanish version of CAMPHOR was used for this study, with permission [17].

### Ethics approval

The study was conducted in compliance with the Good Clinical Practices protocol and the Declaration of Helsinki principles. It was approved by the Ethics Committee of Hospital General Universitario Gregorio Marañón (Madrid, Spain). Completion of CAMPHOR questionnaires was done under the research license of Galen. All patients provided their written informed consent before their inclusion in the study.

### Statistical analysis

Continuous variables are presented as median (interquartile range). Categorical variables are presented as numbers and percentages. The Wilcoxon rank-sum test was used for comparisons between subgroups for CAMPHOR domains (symptoms, activities and quality of life). For continuous variables, the subgroups were formed using median values for each one. The threshold value for 6MWD was determined by calculating the median result of this test in the overall population.

All statistical analyses were performed using Stata-IC version 14 and results were considered significant if p < 0.05.

## Results

### Population characteristics

**Fig. 1:**
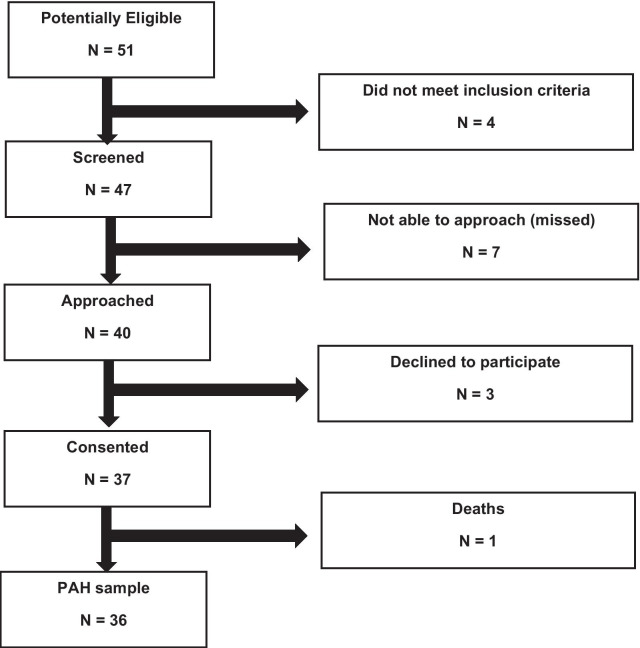
Study flowchart

Of 51 potentially eligible patients, 4 were initially excluded because they did not meet the inclusion criteria: 3 patients had cognitive impairment and 1 patient was not a Spanish speaker (whose questionnaire needed to be read out). Seven patients were not approached because of not having a routine follow-up in the pharmacy consultation or declining to talk with researchers. Five of these seven patients only could complete the questionnaire by telephone and therefore they were excluded. Of 37 patients who gave their signed consent, 1 patient was not included in the final sample because he died during data collection (Fig. 1).

Thirty-six patients consented to participate in the study and completed the questionnaire. All these patients were receiving specific treatment for PH at the time of questionnaire accomplishment. The demographic and clinical characteristics according to WHO FC are shown in Table 1.

**Table 1:** Demographic and clinical characteristics by WHO FC

Characteristics	WHO FC I/II	WHO FC III
	(n = 23)	(n = 13)
Age, years	62.5 (53.0–75.6)	58.9 (52.9–64.3)
Female, n (%)	14 (60.9)	8 (61.5)
Time since diagnosis, years	5.7 (1.6–8.9)	2.0 (1.2–6.7)
PH etiology, n (%)		
PAH		
Congenital heart disease	5 (21.7)	2 (15.4)
Idiopathic	4 (17.4)	–
HIV–associated	4 (17.4)	–
Scleroderma	3 (13.0)	3 (23.1)
Portopulmonary hypertension	3 (13.0)	2 (15.4)
Eisenmenger syndrome	–	3 (23.1)
Drug-associated	1 (4.4)	1 (7.6)
Connective tissue disease, not scleroderma	1 (4.4)	–
CTEPH	2 (8.7)	2 (15.4)
Time undergoing treatment, years	3.2 (1.3–5.9)	1.7 (0.9–2.9)
Type of treatment for PH, n (%)		
PDE-5 is	18 (78.3)	11 (84.6)
ERA	12 (52.2)	7 (53.9)
PC analogs and receptor agonists	2 (8.7)	2 (15.4)
PH specific therapy, n (%)		
Monotherapy	15 (65.2)	6 (46.1)
Double combination therapy	6 (26.1)	5 (38.5)
Triple combination therapy	2 (8.7)	2 (15.4)
Line of treatment, n (%)		
First-line	17 (73.9)	7 (53.8)
Second line	4 (17.4)	4 (30.8)
Third line	–	2 (15.4)
Fourth line	2 (8.7)	–
Number of concomitant drugs	6 (3–8)	7 (4–9)
Number of comorbidities	2 (1–3)	2 (1–4)
Diuretics, n (%)		
Yes	15 (65.2)	11 (84.6)
No	8 (34.8)	2 (15.4)
Oral anticoagulants, n (%)		
Yes	6 (26.1)	3 (23.1)
No	17 (73.9)	10 (76.9)
Oxygen therapy, n (%)		
Yes	2 (8.7)	3 (23.1)
No	21 (91.3)	10 (76.9)
At least 1 emergency visit in the last 12 months, n (%)	7 (30.4)	4 (30.8)
Number of emergency visits in the last 12 months	0 (0–1)	0 (0–1)
At least 1 hospital admission in the last 12 months, n (%)	6 (26.1)	5 (38.5)
Number of hospital admissions in the last 12 months	0 (0–1)	0 (0–1)
6MWD, meters	447.4 (362.3–488.0)	329.4 (296.9–390.0)
SBP_Bas, mmHg	110.0 (110.0–137.5)	110.0 (90.0–125.0)
SBP_Max, mmHg	157.5 (130.0–180.0)	135.0 (120.0–150.0)
Borg dyspnea	3 (2–5)	7 (3–10)
O2Sat_Bas, mmHg	97.0 (96.0–98.0)	95.0 (92.0–96.0)
O2Sat_Min, mmHg	92.0 (88.0–94.0)	92.0 (84.0–92.0)
NT-proBNP, pg/mL	238.0 (122.0–592.0)	487.0 (120.0–1348.0)
mPAP, mmHg	35.0 (23.0–40.0)	34.5 (26.0–52.5)
Cardiac output, L/min	4.2 (3.0–5.2)	4.1 (3.7–5.5)
RAP, mmHg	7.0 (5.0–12.0)	5.5 (4.0–7.0)
PVR, Wood units	4.9 (2.8–6.6)	4.3 (2.1–7.4)

Data displayed as median (interquartile range), except with otherwise indicated; PAH: pulmonary arterial hypertension, PH: pulmonary hypertension, CTEPH: Chronic thromboembolic pulmonary hypertension, PDE-5 is: phosphodiesterase type 5 inhibitors, ERA: endothelin receptor antagonist, PC: prostacyclin, 6MWD: 6-min walking distance, SBP_Bas: baseline systolic blood pressure, SBP_Max: maximum systolic blood pressure, O2Sat_Bas: baseline oxygen saturation, O2Sat_Min: minimum oxygen saturation, NT-proBNP: N-terminal pro-brain natriuretic peptide, mPAP: mean pulmonary arterial pressure, RAP: right atrial pressure, PVR: pulmonary vascular resistance

Most of the patients were receiving a PDE-5 inhibitor in both groups (78.3% and 84.6%), showing similar proportions of patients receiving ERAs (52.2% and 53.9%) and a higher proportion of prostacyclin analogs and receptor agonists in WHO FC III (15.4% vs 8.7%). The number of concomitant drugs and comorbidities per patient was higher in patients in WHO FC III comparing to those in I/II but without statistically significant differences (p = 0.260, p = 0.221).

Regarding clinical variables, significant differences between patients in WHO functional class I/II to III WHO were found for Borg dyspnea (p = 0.043), but 6MWD (p = 0.077). A higher increase in systolic blood pressure was found when performing 6-min walk test in WHO FC I-II, whether a greater arterial oxygen desaturation was found in patients in WHO FC III.

### Factors affecting CAMPHOR scores

**Fig. 2:**
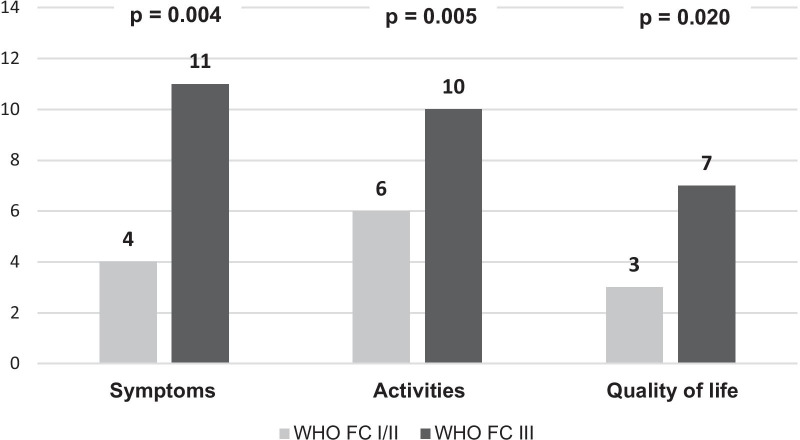
CAMPHOR scores according to WHO Functional Classification

CAMPHOR scores in the different domains showed results that indicated a moderate impairment in the quality of life of patients diagnosed with PH. Median scores for symptoms, activities, and quality of life domains were 5.5 (2.5–10), 8.0 (4.5–10.5) and 3.5 (1–7.5), respectively. When comparing patients who were in WHO FC III to I/II, significant differences were found in all CAMPHOR domains (Fig. 2).

Regarding demographic variables, the median age of 60 years was considered to stratify patients into two subgroups. However, no statistically significant differences were found for age and gender in any of the CAMPHOR domains (Table 2).

**Table 2:** Factors affecting CAMPHOR domains

	Symptoms	p	Activities	p	Quality of life	p
Age						
< 60 years	6.5		6.5		3.0	
≥ 60 years	4.5	0.504	9.0	0.634	4.0	0.763
Gender						
Male	5.5		9.0		4.0	
Female	5.0	0.454	5.0	0.118	2.5	0.289
Time since diagnosis						
< 5 years	5.0		9.0		3.0	
≥ 5 years	6.0	0.691	6.0	0.351	4.0	0.917
Combination therapy						
Yes	6.0		9.0		2.0	
No	4.0	0.784	7.0	0.974	4.0	0.508
Line of treatment						
First line	5.0		6.5		4.0	
Second or later	6.5	0.893	9.0	0.800	2.5	0.399
Number of concomitant drugs						
< 7 drugs	4.0		6.0		3.0	
≥ 7 drugs	7.5	0.040	9.5	0.135	4.0	0.078
Number of comorbidities						
< 3	4.0		6.0		3.0	
≥ 3	8.0	0.155	10.0	0.170	7.0	0.085
Borg dyspnea						
< 4	6.0		6.0		6.0	
≥ 4	8.0	0.262	8.0	0.313	8.0	0.350
At least 1 emergency visit in the last 12 months						
No	4.0		6.0		3.0	
Yes	7.0	0.098	10.0	0.214	7.0	0.045
At least 1 hospitalization in the last 12 months						
No	4.0		6.0		3.0	
Yes	7.0	0.020	10.0	0.063	7.0	0.040

Clinical data showed no statistically significant differences regarding time since diagnosis, use of specific combination therapy and line of treatment. When comparing patients receiving less than 7 vs 7 or more concomitant drugs, a significant difference was found for the symptoms domain (p = 0.040) but it was not maintained across the rest of domains (p = 0.135 and p = 0.078 for activities and quality of life domain, respectively). The three CAMPHOR domains showed greater scores in those patients who had 3 or more comorbidities. Nevertheless, they did not reach statistically significant differences.

**Fig. 3:**
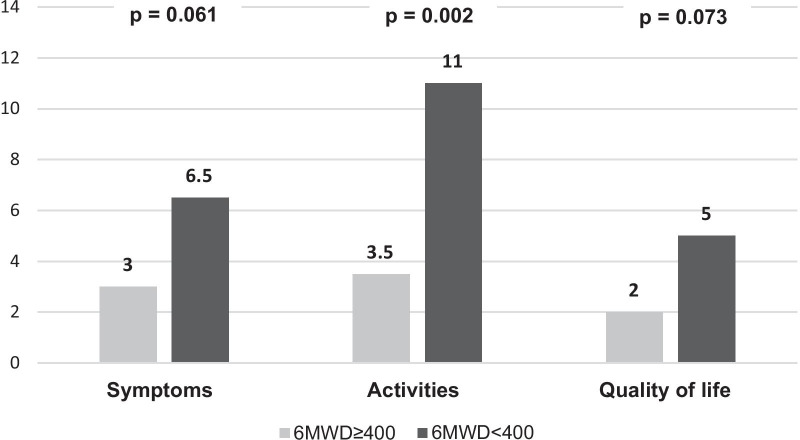
CAMPHOR scores according to 6MWD

Significant differences were found in the quality of life domain when analyzing by patients who had an EV in the last 12 months (3.0 vs 7.0, p = 0.045) as represented. Besides, the comparison between patients who had at least one HA in the last 12 months than those who had not, showed statistically significant differences in symptoms and quality of life domains (p = 0.020 and p = 0.040, respectively) but not in the activities domain (p = 0.063). When comparing patients who had a result above and below 400 m in the 6-m walking test, higher scores were found in the subgroup who walked less than 400 m. The differences reached statistically significance for the activities (p = 0.002) but not for the symptoms (p = 0.061) and quality of life (p = 0.073) domain (Fig. 3). Finally, the CAMPHOR scores when stratifying by Borg dyspnea scale were higher for those scoring 4 or more, without statistically significant differences.

## Discussion

Our study explored the clinical characteristics, HRQoL, and QoL of a Spanish cohort of patients diagnosed with PH. All the patients included were receiving specific treatment for PH, which was dispensed at the pharmacy consultation, and were routinely monitored.

Generic HRQoL measures employed in PH population such as the NHP, EuroQoL and SF-36 have proved to be of limited value in the assessment of PH because they are not specific for symptoms and limitations associated to this disease. CAMPHOR was developed to be a disease-specific and practical QoL instrument in these patients, using unidimensional subscales that are reproducible and valid [14]. For those reasons, it was the selected questionnaire for this study. To our knowledge, this is the first study performed in Spain using CAMPHOR as a direct measure of PRO´s. Besides, it was the first protocol which used this questionnaire in a pharmacy consultation, whereas the clinicians had the information for their routine visits.

After completion of CAMPHOR, the results obtained in the three domains show that our attended PH population (WHO FC I-III) experiences different levels of impairment due to disease. Our results were slightly lower than those reported by Reis et al. [5] in a similar cohort of patients (69.4% in WHO FC I/II vs 63.9% in our study) and reasonably lower than those reported by McCabe et al. for patients with idiopathic PAH and CTEPH) [18]. Regarding factors that could affect these results and consistent with those reported by Small et al. [19], in the three CAMPHOR domains the scores were better in those patients receiving only one drug since 65.2% of WHO FC I/II and 46.2% of WHO FC III patients in our study were undergoing monotherapy for PH. This could explain the lower scores obtained.

The non-parametric analysis of clinical characteristics and HRQoL performed in this study showed that WHO FC and 6MWD were the two items that had better relations with CAMPHOR scores. On the one hand, the WHO FC remains one of the most powerful predictors of survival during diagnosis and follow-up, and its worsening is an indicator of disease progression [2]. In our study, approximately a third of patients were in WHO FC III and no patient was found to be in WHO FC IV. These proportions were higher than those reported in the REVEAL registry [20] and previous studies with measures of HRQoL that show proportions of WHO FC III-IV near 70% [18, 19, 21]. The association between WHO FC and CAMPHOR scores has been well described in previous cohorts [5]. We found statistically significant differences for the WHO FC and the `Symptoms´ (p = 0.004), `Activities´ (p = 0.005) and `Quality of life´ (P = 0.02) domains of CAMPHOR, showing that PRO´s are good predictors of the functional capacity.

On the other hand, 6MWD is usually used to stratify the risk of PAH patients and to assess the effectiveness of PAH specific therapies [10]. A worsening value directly indicates a decrease in the functional capacity. We found worse scores of the 6MWD for patients in WHO FC III, however, they did not reach statistically significant differences (p = 0.077). In the non-parametric analysis, the differences for this test were significant for the `Activities´ domain (p = 0.002), as reported elsewhere [22] using a generic questionnaire in PH patients (SF-36) proving that the `physical´ component score was the one that obtained statistically significant differences. This parameter has proved to be superior to other parameters such as Borg dyspnea in the clinical evaluation of patients with PH.

We also found worse scores in the `Quality of life´ domain for patients who had at least one emergency visit in the previous 12 months and in the `Symptoms´ and `Quality of life´ domains for those who had been hospitalized in the last 12 months. Recent hospitalizations have been previously reported to correlate with impaired quality of life [19, 23] due to the association between the need for in-patient care with the severity of disease and other possible complications. These differences were not maintained across the three CAMPHOR domains probably due to the small sample size.

The main limitations of our study derive from the small number of patients included, but due to the low prevalence of this disease in the overall population (15–52 cases per million people), it is difficult to recruit big samples [8]. Some non-parametric analyses were likely to be significant if more patients had been included. Also, patients in our setting can be treated in different hospitals and because of that, the real proportion of this disease could be underestimated. Our centre does not perform lung or thromboendarterectomy. Therefore, patients with more advanced disease are usually recruited to other hospitals and because of that most of our patients belong to WHO FC I and II. Besides, some patients needed to be excluded from the study because we were not able to approach them or because they declined to participate. More patients would need to be assessed to have a full spectrum of PH. Finally, the CAMPHOR questionnaire, as a disease-specific instrument, was the only one performed in our study.

CAMPHOR has proved to be a specific measure of the clinical condition (symptoms and activities domains) and the impact of PH from the patient´s perspective (quality of life domain), as their scores are significantly different for patients with a worse WHO FC and recent hospitalizations. The clinical evaluation in these patients is performed using risk assessment predictors (WHO FC and 6MWD). However, they only represent one aspect of functioning, whereas the CAMPHOR scale covers wider activities of daily living and it has proved to be correlated with these two measures. Because of that, the PRO´s are highly useful in this type of patients who cannot be correctly evaluated through the other measures, as they don´t capture the full impact of the disease. Also, CAMPHOR questionnaire reports information about symptoms and burden of disease, not only functional capacity.

In some cases, and given the good relation with these measures, these questionnaires could replace invasive or unpleasant tests such as RHC or 6-min walk. The PRO´s reported in CAMPHOR are useful tools to complete clinical evaluation in these patients which is difficult for the need of using other measures (6MWD, WHO FC, Borg dyspnea) which are difficult to perform.

## Conclusions

In conclusion, the CAMPHOR questionnaire could be useful as a complementary test to achieve an integrated evaluation of PH patients. Their relations with traditional measures used in these patients and its wide coverage of all factors (symptoms, functional capacity and burden of disease) which could affect the clinical condition are important issues to establish this quality of life test as a routine evaluation for PH. CAMPHOR questionnaire was easily completed by patients during their routine pharmacy visits, with good acceptance.

These questionnaires could be performed by other health professionals (using information and communication technologies when possible), and their results could be incorporated to the patient´s electronic medical record to be available for the cardiologist´s follow-up visit. This assessment is easy to implement in any institution and could help improve the evaluation and health outcomes of these patients.

## Data Availability

The datasets generated used and analysed during the current study are available from the corresponding author on reasonable request.

## Abbreviations

PAH: Pulmonary arterial hypertension
PH: Pulmonary hypertension
PAPm: Resting pulmonary arterial pressure
LHD: Left heart disease
CTEPH: Chronic thromboembolic pulmonary hypertension
WHO FC: World Health Organization functional class
6MWD: 6-M walking distance
NT-proBNP: N-terminal prohormone of brain natriuretic peptide
CI: Cardiac index
RAP: Right atrial pressure
QoL: Quality of life
HRQoL: Health-related quality of life
PDE-5 is: Phosphodiesterase type 5 inhibitors
ERA: Endothelin-receptor antagonists
SGCs: Soluble guanylate cyclase stimulators
PCa: Prostacyclin analogs
PCRa: Prostacyclin receptor agonists
AE: Adverse events
PRO´s: Patient reported outcomes
CAMPHOR: Cambridge Pulmonary Hypertension Outcome Review
NHP: Nottingham Health Profile
EV: Emergency visits
HA: Hospital admissions
RHC: Right heart catheterization
CPOE: Computerized Physician Order Entry
